# Transcriptomic Insights and Cytochrome P450 Gene Analysis in *Kadsura coccinea* for Lignan Biosynthesis

**DOI:** 10.3390/genes15030270

**Published:** 2024-02-21

**Authors:** Hanyu Fu, Chuan Guo, Jiqing Peng, Fengxia Shao, Song Sheng, Sen Wang

**Affiliations:** 1 College of Forestry, Central South University of Forestry & Technology, 498 South Shaoshan Road, Changsha 410004, China; fuhanyu0411@163.com (H.F.); 15116278764@163.com (C.G.); pengjiqing17@csuft.edu.cn (J.P.); shaofx1103@163.com (F.S.); 2 Yuelushan Laboratory, Qiushi Building, Hunan Agricultural University, Furong District, Changsha 410128, China; 3 The Belt and Road International Union Research Center for Tropical Arid Non-Wood Forest in Hunan Province, 498 South Shaoshan Road, Changsha 410004, China

**Keywords:** transcriptome, Cytochrome P450, phylogenetic analysis, lignan biosynthesis, *Kadsura coccinea*, methylenedioxy bridges, pharmacology

## Abstract

*Kadsura coccinea* is a medicinal plant from the Schisandraceae family that is native to China and has great pharmacological potential due to its lignans. However, there are significant knowledge gaps regarding the genetic and molecular mechanisms of lignans. We used transcriptome sequencing technology to analyze root, stem, and leaf samples, focusing on the identification and phylogenetic analysis of Cytochrome P450 (CYP) genes. High-quality data containing 158,385 transcripts and 68,978 unigenes were obtained. In addition, 36,293 unigenes in at least one database, and 23,335 across five databases (Nr, KEGG, KOG, TrEMBL, and SwissProt) were successfully annotated. The KEGG pathway classification and annotation of these unigenes identified 10,825 categorized into major metabolic pathways, notably phenylpropanoid biosynthesis, which is essential for lignan synthesis. A key focus was the identification and phylogenetic analysis of 233 Cytochrome P450 (CYP) genes, revealing their distribution across 38 families in eight clans, with roots showing specific CYP gene expression patterns indicative of their role in lignan biosynthesis. Sequence alignment identified 22 homologous single genes of these CYPs, with 6 homologous genes of CYP719As and 1 of CYP81Qs highly expressed in roots. Our study significantly advances the understanding of the biosynthesis of dibenzocyclooctadiene lignans, offering valuable insights for future pharmacological research and development.

## 1. Introduction

**Figure 1:**
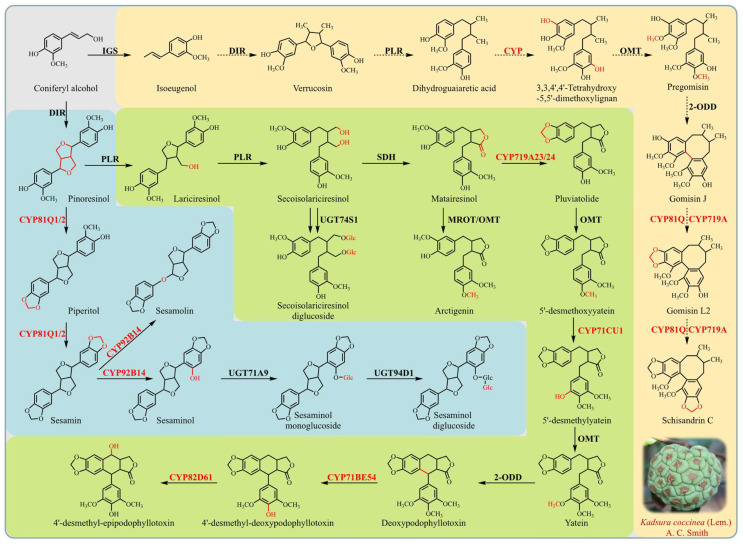
Biosynthesis pathways of major lignans in *K. coccinea*. Note: The schematic diagram illustrates the biosynthetic pathways leading to the formation of major lignans in *K. coccinea*. CYPs involved in these pathways are highlighted in red. Solid and broken lines represent identified and unidentified enzyme-catalyzed reactions, respectively. Enzymes involved in the pathways include the following: IGS, isoeugenol synthase; DIR, dirigent; PLR, pinoresinol–lariciresinol reductase; CYP, cytochrome P450; OMT, *O*-methyltransferase; SDH, secoisolariciresinol dehydrogenase; UGT, UDP-glucose-dependent glucosyltransferase; 2-ODD, 2-oxoglutarate/Fe(II)-dependent dioxygenase; MOMT, matairesinol *O*-methyltransferase.

*K. coccinea*, a member of the Schisandraceae family, is predominantly found in several provinces of China, including Jiangxi, Hunan, Sichuan, and Yunnan. Due to its wide array of pharmacological properties, such as improving liver function [1,2,3,4], anti-inflammation [5,6], anti-tumor [7], anti-HIV [8], anti-oxidation [9], anti-coagulation [10], and so on [11,12], this plant has gained considerable attention in the field of traditional medicine. The primary bioactive constituents of *K. coccinea* are lignans, with more than 121 types identified. These include predominantly dibenzocyclooctadiene lignans, along with lesser quantities of arylnaphthalene and dibenzylbutane lignans [13,14]. The biosynthetic pathway of lignans is the phenylpropanol pathway, which is formed by the oxidation polymerization of two phenylpropane units. This process is affected by a series of enzyme modifications and produces a variety of lignan structures [15]. Key enzymes involved in this process include dirigent (DIR), pinoresinol–lariciresinol reductase (PLR), secoisolariciresinol dehydrogenase (SDH), *O*-methyltransferase (OMT), cytochrome P450 (CYP), and UDP-glucose-dependent glucosyltransferase (UGT) (Figure 1). These enzymes have been identified in various plant species, such as *Podophyllum peltatum* [16], *Forsythia suspensa* [17], and *Sesamum indicum* [18], underscoring the widespread nature of lignan biosynthesis in the plant kingdom.

In terms of lignin biosynthesis regulation, Behr et al. [19] provided valuable insights into the molecular regulation of monolignol-derived product biosynthesis in hemp, indicating the involvement of dirigent and dirigent-like proteins in this process. Additionally, Dar and Arumugam [20] conducted a comprehensive review of the biosynthesis of sesame lignans, highlighting not only their health benefits, but also the metabolic pathways involved in their formation. Kim et al. [21] demonstrated the feasibility of the metabolic engineering of lignan biosynthesis in *Forsythia* cell cultures, showcasing the potential of producing beneficial lignans in plant cell cultures. Satake et al. [22] have discussed the potential of metabolic engineering in enhancing lignan production, emphasizing the criticality of a thorough understanding of lignan biosynthetic pathways for efficient production. Ražná et al. [23] explored the role of microRNAs in the regulation of lignan biosynthesis, suggesting a complex and intricate regulatory network at the molecular level. These studies in the field have significantly deepened our understanding of lignan biosynthesis.

CYP450 enzymes are currently recognized as the most expansive enzyme family in plant metabolism, playing a pivotal role in the biosynthesis of a diverse array of natural products, encompassing terpenoids, alkaloids, and phenylpropanoids [24]. These enzymes are essential for producing complex phytochemicals, enhancing the chemical diversity within the plant kingdom, and contributing to the synthesis of pharmacologically active compounds. Key CYP450 enzymes such as C4H (CYP73A) and C3H (CYP98A) are instrumental in the formation of the lignan precursor coniferyl alcohol, a process detailed by Ehlting et al. [25]. The biosynthetic pathway of 4′-desmethyl-epipodophyllotoxin, an important precursor to the anticancer drug etoposide, is marked by the involvement of several CYP450 enzymes. Notably, CYP719A23 is responsible for catalyzing the formation of the methylenedioxy bridge in pluviatolide—a crucial step in this pathway [16]. Other CYP450 enzymes, such as CYP71CU1, CYP71BE54, and CYP82D61, are involved in further modifications, leading to podophyllotoxin analogs [26]. In *S. indicum*, the enzymes CYP81Q1/2 and CYP92B14 are instrumental in the biosynthesis of sesamin and its derivatives, showcasing the diverse functions of CYPs in lignan formation [27,28] (Figure 1). Additionally, the biosynthesis of dibenzocyclooctadiene lignans in Schisandraceae plants starts with isoeugenol conversion, involving a series of reactions catalyzed by DIR, PLR, CYP, OMT, and UGT enzymes, leading to complex lignan structures [29]. However, the complete biosynthetic pathway of these lignans is yet to be fully elucidated.

Previous studies on the accumulation sites of lignans have been conducted. Liang et al. [30] conducted an in-depth metabolomic and transcriptomic analysis, uncovering a pronounced presence of lignans in the root tissues. This finding is complemented by the work of Liu and Li [31], who isolated lignans from the seeds of the plant, indicating their widespread distribution, albeit without a direct comparison to root concentrations. Additionally, Fang et al. [32] corroborated the presence of lignans in the roots, aligning with the observations made by Liang et al. [30]. In this context, we use transcriptome analysis—a basic method of plant functional genomics—to focus on the study of lignan-rich tissues and organs, systematically screen and identify all CYP genes, and reveal the different distribution of these genes in different clans, with special emphasis on clans that are indispensable for lignan biosynthesis. This comprehensive transcriptomic analysis, combined with CYP gene identification, establishes a robust foundation for further investigation of the lignan biosynthetic pathway in *K. coccinea*, thereby enhancing our understanding of this medicinally significant plant.

## 2. Materials and Methods

### 2.1. Plant Materials

*K. coccinea* samples were collected from the *K. coccinea* Chinese herbal medicine cooperative in Tongdao County, Hunan Province. Specimens, encompassing roots, stems, and leaves, exhibiting robust growth and free from pests and diseases were selected and collected over a four-year period. Each plant part was represented by three biological replicates. Immediately after collection, samples were flash-frozen in liquid nitrogen and stored at −80 °C for subsequent analysis.

### 2.2. Transcriptome Library Construction and Sequencing

Total RNA served as the starting material for library construction. mRNA with polyA tails was enriched using Oligo(dT) magnetic beads. The mRNA was then fragmented using Fragmentation Buffer. This fragmented mRNA served as a template for first-strand cDNA synthesis using random oligonucleotides as primers. The synthesis of the second cDNA strand involved the addition of buffer, dNTPs, DNA polymerase I, and RNaseH. The resulting double-stranded cDNA was purified, end-repaired, A-tailed, and ligated with sequencing adapters. Fragment size selection was conducted using AMPure XP system, followed by PCR amplification. The PCR products were further purified using AMPure XP system (Beckman Coulter, Beverly, Pasadena, CA, USA) to obtain the final library. The library was initially quantified using a Qubit2.0 Fluorometer, diluted to 1.5 ng/µL, and its insert size was assessed using an Agilent 2100 bioanalyzer (Agilent Technologies, Santa Clara, CA, USA). Following confirmation of the insert size, the library’s effective concentration was accurately quantified using qRT-PCR to ensure quality. Sequencing was performed using the Illumina NovaSeq 6000 platform by Novogene Co., Ltd. (Tianjin, China).

### 2.3. Transcriptome Data Assembly and Gene Annotation

Raw data from sequencing were processed using Trimmomatic (version 0.39) to remove adapters, reads containing undetermined bases (N), and low-quality reads [33]. Clean reads were then assembled de novo using Trinity (version 2.14.0) software [34]. Redundant sequences in the transcripts were eliminated using RapClust (version 0.1.2) software to yield unigenes [35]. The CDS regions of unigenes were predicted using TransDecoder (version 5.5.0) software. The predicted CDSs were aligned against local databases (Nr, KEGG, KOG, TrEMBL, and SwissProt) using DIAMOND (version 2.0.15) software to obtain annotation information [36]. The sequence with the best alignment was selected for final annotation. The PfamScan (version 1.6) program annotated all unigene sequences in the Pfam database (version 35.0), selecting sequences with E values lower than 1 × 10^−5^ for final structural annotation. GO and KO annotations were performed using InterProScan (version 5.59–91.0) and KofamScan (version 1.3.0), respectively [37,38]. GO and KEGG histograms were generated using the ggplot2 (version 3.3.5) package in R (version 4.1.1), and the Venn diagram was created using the BioLadder bioinformatics online analysis website.

### 2.4. Screening and Phylogenetic Tree Construction of CYP Genes in K. coccinea

CYP (PF00067) annotation information was extracted from the Pfam results, and sequences shorter than 280 amino acids were discarded. The protein sequences of *Arabidopsis thaliana* CYPs were downloaded for reference. Multiple sequence alignment of *K. coccinea* CYP sequences with those of *A. thaliana* was performed using ClustalW. Phylogenetic trees were constructed using MEGA 11.0 software, employing the maximum likelihood (ML) method with a bootstrap value set to 1000. CYP sequences were classified into gene families and subfamilies based on international nomenclature standards, considering amino acid sequence homology thresholds of 40% and 55%, respectively.

### 2.5. Screening of Candidate CYP Genes Related to Lignan Biosynthesis in K. coccinea

Expression levels of *K. coccinea* unigenes in roots, stems, and leaves were calculated using Salmon (version 1.9.0) software [39]. Heat map clustering analysis was performed using the Pheatmap (version 1.0.12) package in R (version 4.1.1), normalizing data rows before clustering. BlastP alignment was conducted between CYP protein sequences related to lignan biosynthesis in other species and all identified CYP family protein sequences in *K. coccinea*. Candidate members were aligned and phylogenetic trees were constructed using MEGA11.0 software. Branches closely related to reported members were selected for further analysis and tree reconstruction using MEGA11.0 software. The tree files were refined using the iTOL online website, and the heat map of the phylogenetic tree was created similarly.

### 2.6. Quantitative Real-Time PCR Analysis

To verify the accuracy of gene expression levels in the transcriptome data, we randomly selected 8 genes from the identified cytochrome P450 (CYP) genes for qRT-PCR analysis, with Actin as the internal reference. Specific primers for qRT-PCR were designed using Primer software (version 5) (Table S1). The qPCR reaction system was prepared according to the instructions of Sigma’s 2 × SYBR Green Master Mix Enzyme kit. Amplification proceeded as follows: pre-denaturation at 95 °C for 5 min, 40 cycles at 95 °C for 15 s, annealing at 60 °C for 40 s. Dissolution curves were recorded from 60 °C to 95 °C, with a 0.5 °C increase every 5 s. Each sample had three biological replicates. Relative expression levels of target genes were calculated using 2^−ΔΔCq^.

## 3. Results

### 3.1. High-Quality Transcriptome Sequencing and Assembly of K. coccinea

**Figure 2:**
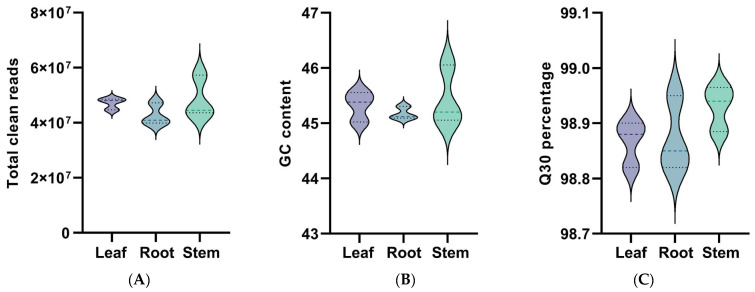
Quality assessment of RNA-Seq sequencing data. The labels L, R, and S denote leaf, root, and stem tissues, respectively. (**A**) Total Clean Reads—this violin plot compares the number of clean reads obtained from the sequencing of leaf, root, and stem samples of *K. coccinea*. (**B**) GC Content—this plot displays the distribution of guanine–cytosine (GC) content in the sequences derived from different tissue samples. (**C**) Q30 Percentage—this plot illustrates the percentage of sequences with a quality score above 30, indicative of a very low probability of sequencing errors. The *x*-axis represents the sample types (leaf, root, stem), and the *y*-axis denotes the respective measures (total reads, GC content, Q30 percentage). The shape of each violin plot reflects the variability and density of data points.

To delve into the molecular basis of its pharmacological properties through transcriptome sequencing, rigorous quality control measures were applied to the transcriptome sequencing data of nine samples (root, stem, leaf). The sequencing results yielded a substantial number of clean reads across different plant parts, demonstrating high-quality data acquisition (Figure 2). Specifically, leaf samples produced 47,098,468 clean reads, with a GC content of 45.32% and an impressive average Q30 score of 98.87%. Root samples contributed 42,677,773 clean reads, featuring a GC content of 45.17% and maintaining the same high average Q30 of 98.87%. Stem samples generated 48,450,287 clean reads, with a slightly higher GC content of 45.44% and an average Q30 of 98.93%. These statistics underscore the excellent quality of the library’s construction and the reliability and accuracy of the sequencing data. The transcriptome assembly and clustering process resulted in the identification of 158,385 transcripts. These transcripts exhibited an average length of 1052 base pairs (bp), a GC content of 41.59%, and an N50 value of 1748 bp. Subsequent steps to eliminate redundant sequences led to the isolation of 68,978 unigenes. These unigenes had an average length of 1049 bp, a GC content of 41.85%, and an N50 of 1644 bp. These metrics indicate a high-quality assembly of the *K. coccinea* transcriptome sequence, providing a robust foundation for further genomic analysis and research.

### 3.2. Annotation of Unigenes in K. coccinea with Multi-Step Bioinformatics Tools

**Figure 3:**
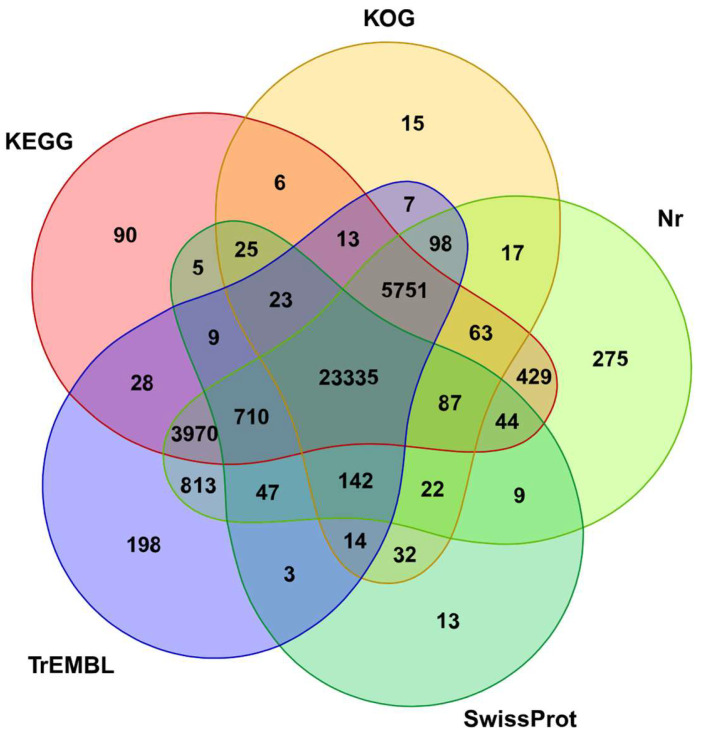
Venn diagram of gene annotation across databases. Each circle in this Venn diagram represents a specific database. The overlapping areas show the number of genes that are commonly annotated across multiple databases, while the unique numbers outside the overlaps indicate genes uniquely annotated in each respective database.

The process of annotating unigenes in this study involved several steps, utilizing advanced bioinformatics tools. Initially, the TransDecoder (version 5.5.0) was employed to predict the coding sequence (CDS) regions of the unigenes. Following this, the predicted CDS were subjected to a BLAST analysis using DIAMOND software (version 2.0.15). This analysis was conducted against a suite of local databases, including Nr, KEGG, KOG, TrEMBL, and SwissProt. The sequences that exhibited the best alignment were selected as the final annotation sequences. The results of the annotation process are summarized in Table S2. A significant proportion of unigenes, 36,293 (52.62%), were successfully annotated in at least one of the databases. Furthermore, 23,335 unigenes (33.83%) were annotated across all five databases, as illustrated in Figure 3. The Nr database emerged as the most comprehensive source for unigene annotation, with 35,812 unigenes (51.92% of the total annotated genes) being annotated in this database. The TrEMBL database followed closely, annotating 35,161 unigenes, which accounts for 50.97% of the annotated unigenes. In contrast, the SwissProt database had the lowest number of annotated unigenes, with 24,520 unigenes (35.55%) being annotated. This comprehensive annotation process provides a valuable resource for understanding the functional genomics of *K. coccinea* and lays the groundwork for further genetic and biochemical studies.

### 3.3. Elucidating the Molecular Functions and Metabolic Pathways in K. coccinea through Gene Ontology and KEGG Analysis

**Figure 4:**
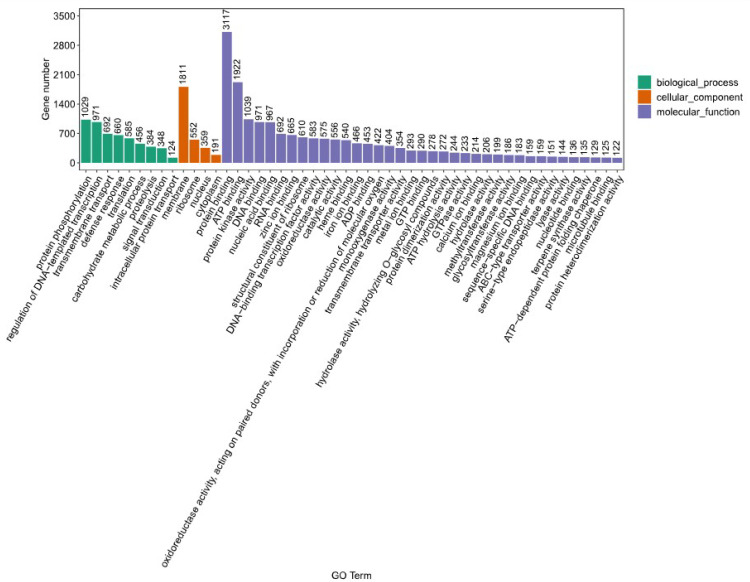
Distribution of Gene Ontology (GO) categories. The bar chart displays the distribution of genes across various Gene Ontology (GO) terms. The *x*-axis lists the GO terms, and the *y*-axis shows the number of genes associated with each term. Different colors are used to differentiate between the GO categories.

To understand the plant’s intricate biological processes and molecular interactions, the Gene Ontology (GO) functional annotation was meticulously performed using the Pfam database (version 35.0) through PfamScan (version 1.6) and InterProScan (version 5.59–91.0) software. This process successfully annotated a total of 19,973 unigenes. Within the biological process category, 13,747 unigenes were annotated, with protein phosphorylation being the most represented process, involving 1029 unigenes. In the cellular component category, membrane components were predominant, with 1811 unigenes annotated. The molecular function category saw the highest annotation in protein binding, with 3117 unigenes. These annotations, as illustrated in Figure 4, provide a detailed overview of the functional aspects of the unigenes, highlighting the diverse biological activities and molecular functions present in *K. coccinea*.

**Figure 5:**
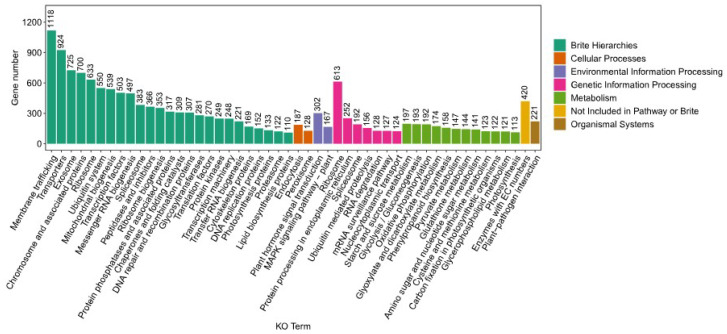
Representation of KEGG pathway categories. The bar chart illustrates the categorization of genes within various Kyoto Encyclopedia of Genes and Genomes (KEGG) pathways (KO Terms). The *x*-axis lists the KEGG pathways, while the *y*-axis indicates the number of genes involved in each pathway. The color coding represents different functional categories such as metabolism, genetic information processing, etc.

Complementing the GO annotation, the KEGG (Kyoto Encyclopedia of Genes and Genomes) pathway classification and the annotation of *K. coccinea* unigenes were meticulously performed using KofamScan software (version 1.3.0). This process involved categorizing the unigenes based on the metabolic pathways they are involved in, as depicted in Figure 5. A total of 10,825 unigenes were successfully annotated and classified into six major metabolic pathway categories. The category with the highest number of annotated unigenes was ‘Brite Hierarchies’, with 10,179 unigenes. In contrast, the ‘Organismal Systems’ category had the lowest number of annotated unigenes, totaling only 221. The unigenes annotated in the ‘Cellular Processes’, ‘Environmental Information Processing’, and ‘Genetic Information Processing’ categories numbered 315, 469, and 1592, respectively. Additionally, 1825 unigenes were annotated under the ‘Metabolism’ category. Focusing on the specific metabolic pathways relevant to *K. coccinea*, it was found that 158 unigenes participate in phenylpropanoid biosynthesis. Specifically, 20 unigenes were associated with sesquiterpenoid and triterpenoid biosynthesis, 7 with monoterpenoid biosynthesis, 12 with the biosynthesis of diterpenoid biosynthesis, and 72 with terpenoid backbone biosynthesis. In conclusion, this annotation serves as a valuable resource for deciphering the plant’s complex metabolic networks and forms a foundation for future pharmacological research.

### 3.4. Elucidating the Role of Cytochrome P450 Genes in Lignan Biosynthesis of K. coccinea

**Figure 6:**
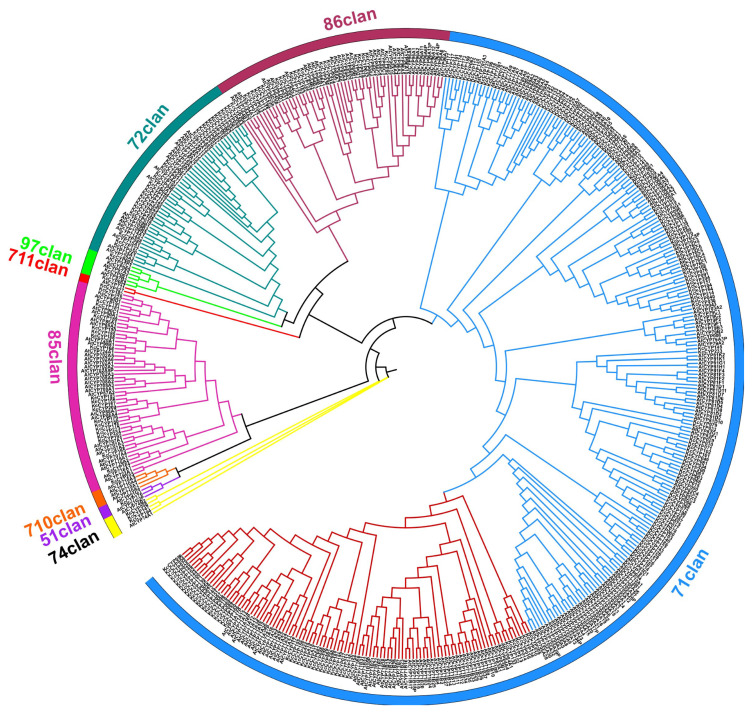
Phylogenetic tree of CYPs in *K. coccinea* and *A*. *thaliana.* The phylogenetic tree (maximum likelihood method) depicts the evolutionary relationships between CYPs in *K. coccinea* and *A. thaliana*. The tree’s structure, with the root at the center, illustrates the genetic or evolutionary distances between the species or sequences.

**Figure 7:**
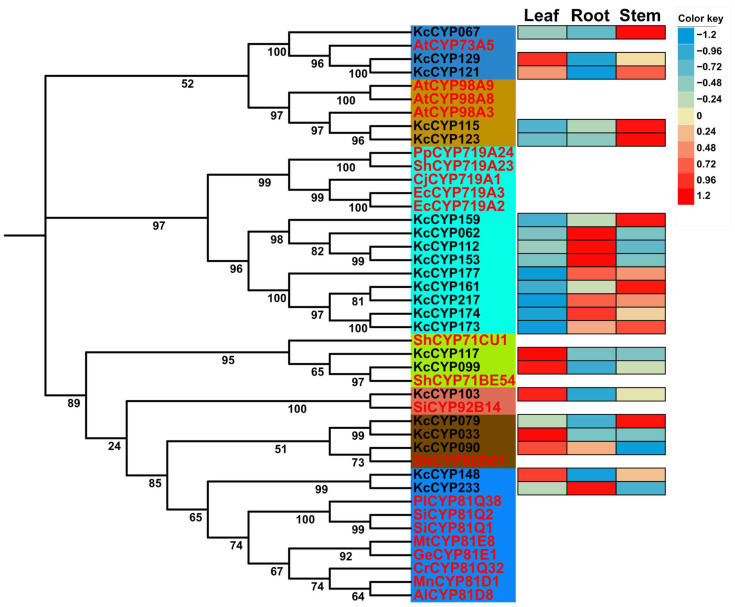
Phylogenetic analysis of CYPs associated with lignan synthesis in *K. coccinea*. The figure presents a phylogenetic analysis of CYPs implicated in lignan biosynthesis in *K. coccinea*. The heatmap overlay indicates the relative expression levels of these enzymes in different tissues, with blue representing lower and red representing higher expression levels. Amino acid sequences were obtained from NCBI GenBank with the following accession numbers: CYP73A5, NP_180607, *A. thaliana*; CYP98A3, OAP09214, *A. thaliana*; CYP98A8, OAP19075, *A. thaliana*; CYP98A9, OAP16063, *A. thaliana*; CYP81Q1, BAE48234, *S. indicum*; CYP81Q2, BAE48235, *S. indicum*; CYP81Q32, AHK60837, *Catharanthus roseus*; CYP81Q38, BAP46307, *Phryma leptostachya*; CYP81D1, EXB59542, *Morus notabilis*; CYP81D8, XP_002866952, *Arabidopsis lyrata* subsp. *lyrata*; CYP81E1, P93147, *Glycyrrhiza echinata*; CYP81E8, AAQ20042, *Medicago truncatula*; CYP92B14, BBB06441, *S. indicum*; CYP719A1, Q948Y1, *Coptis japonica*; CYP719A2, ACO90219, *Eschscholzia californica*; CYP719A3, BAD98249, *E. californica*; CYP719A23, AGC29953, *Sinopodophyllum hexandrum*; CYP719A24, AGC29954, *P. peltatum*; CYP71CU1, ALG05134, *S. hexandrum*; CYP71BE54, ALG05141, *S. hexandrum*; CYP82D61, AGC29951, *S. hexandrum*.

In an effort to understand the metabolic capabilities of *K. coccinea*, particularly in relation to lignan biosynthesis, a comprehensive identification and phylogenetic analysis of cytochrome P450 (CYP) genes was conducted. Utilizing PfamScan software (version 1.6), 233 CYP genes, each annotated with PF00067 and exceeding 280 amino acids in length, were identified and provisionally labeled as KcCYP001 to KcCYP233. A phylogenetic tree, comparing KcCYP protein sequences with those of *A. thaliana* (AtCYPs), revealed that these genes spanned 38 families across eight CYP clans, predominantly distributed across the CYP71 clan, CYP72 clan, CYP85 clan, and the CYP86 clan, with no representation in the 710 clan (Figure 6). Furthermore, root tissues of *K. coccinea*, known for their high lignan concentration, were analyzed for the relative expression levels of all KcCYPs. The analysis showed that 26.6% (62/233), 31.8% (74/233), and 39.1% (91/233) of these genes were relatively highly expressed in the roots, stems, and leaves, respectively (Figure S1). Notably, six genes were silenced across all tissues. Specific expression patterns were observed: *KcCYP061* and *KcCYP153* in the root; *KcCYP087* and *KcCYP150* in the leaves; and *KcCYP006*, *KcCYP007*, *KcCYP118*, *KcCYP179*, and *KcCYP232* in the stem. These findings suggest potential candidate CYP genes involved in lignan synthesis in *K. coccinea*. Additionally, we also reviewed CYPs known to be involved in lignan biosynthesis, including C4H (CYP73A) and C3H (CYP98A) in the biosynthesis pathway of the lignan precursor coniferyl alcohol; CYP719A23/24, CYP71CU1, CYP71BE54, and CYP82D61 in the 4′-desmethyl-epipodophyllotoxin biosynthetic pathway; and CYP81Q1/2 and CYP92B14 in the synthesis of sesamin and sesamolinol. Sequence alignment analysis identified 22 homologous single genes of these CYPs, with differential expression analysis indicating that 6 homologous genes of CYP719As and 1 homologous gene of CYP81Qs were highly expressed in roots (Figure 7). These seven CYP genes are likely involved in the biosynthesis of lignans in *K. coccinea*, marking a significant step forward in biochemical and pharmacological research.

### 3.5. Validation of RNA-Seq Date by qRT-PCR

**Figure 8:**
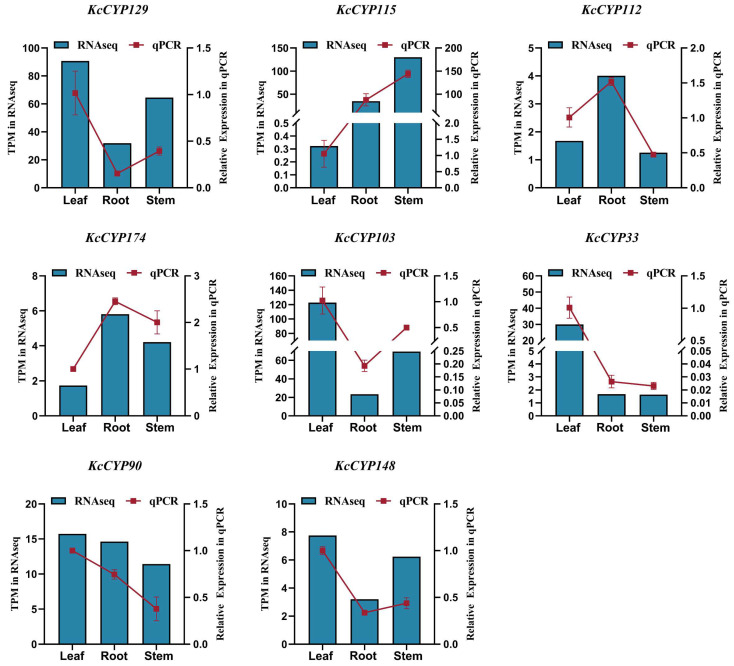
The qRT-PCR verification of eight genes randomly selected from the identified genes. The *Actin* in *K. coccinea* was used as a references gene for qRT-PCR. The relative expression levels of the eight genes were derived from 2^−ΔΔCq^.

The qRT-PCR analysis is an effective method to verify the accuracy of RNA-Seq data. In order to verify the accuracy of our RNA-Seq data, eight genes, including *KcCYP129*, *KcCYP115*, *KcCYP112*, *KcCYP174*, *KcCYP103*, *KcCYP33*, *KcCYP90*, and *KcCYP148*, were randomly selected from the identified genes for qRT-PCR analysis, with *Actin* as the reference gene. The results are shown in Figure 8. The expression trends of the eight genes, obtained through qRT-PCR analysis of the leaves, roots, and stems of *K. coccinea*, are highly consistent with the results of RNA-Seq. These results further enhance the accuracy of our RNA-Seq data.

## 4. Discussion

The investigation into lignan biosynthesis in *K. coccinea* holds considerable significance due to the notable medicinal properties of these compounds [40,41]. Lignans, recognized for their therapeutic potential, are at the forefront of research on *K. coccinea*, a plant esteemed for its pharmacological benefits. In this vein, transcriptome analysis has emerged as a crucial methodology for systematically exploring metabolic pathways in medicinal plants. This approach provides profound insight into the functional genes within the biosynthetic pathways of active ingredients. Recent studies on *Eucommia ulmoides*, *Scutellaria baicalensis*, and *Veratrum mengtzeanum* have made substantial contributions to our understanding of the genetic foundations of pharmacologically active compounds in these plants. Ouyang et al. [42] conducted research on *E. ulmoides*, utilizing transcriptome analysis to identify genes involved in glycoside biosynthesis, thereby illuminating the genetic basis of the plant’s therapeutic properties. In a similar vein, Gao et al. [43] examined *S. baicalensis*, a plant renowned for its flavonoid content. Their transcriptome sequencing efforts successfully identified genes responsible for flavonoid biosynthesis, offering insight into the molecular mechanisms of these compounds, known for their antioxidant and anti-inflammatory properties. Extending this line of inquiry, Liu et al. [44] applied both transcriptome and metabolome profiling to *V. mengtzeanum*, revealing essential elements of the biosynthesis of alkaloids, a class of compounds with a wide range of pharmacological effects. These investigations collectively underscore the utility of transcriptome analysis in revealing the genetic mechanisms behind the synthesis of bioactive compounds in medicinal plants. However, the transcriptome analysis encountered challenges due to the genetic obscurity of the species and the absence of a reference genome. These factors, coupled with potential limitations in sequencing technologies, resulted in approximately 50% of the unigenes remaining unannotated. Our research successfully yielded 158,385 transcripts and 68,978 unigenes. Functional annotation of these unigenes revealed that 36,293 were annotated in at least one public database, marking a significant step in understanding the plant’s genetic makeup. Through KEGG functional annotation, 158 unigenes were implicated in phenylpropanoid biosynthesis, a pathway integral to lignan synthesis that is of particular interest due to the pharmacological importance of lignans.

Cytochrome P450s (CYPs) are the most extensive enzyme family involved in plant metabolism, with about 5100 plant CYP sequences annotated and named to date [45]. In terrestrial plants, the CYP family is categorized into 11 clans, comprising both single-family and multi-family clans [24]. The single-family clans include the CYP51 clan, CYP74 clan, CYP97 clan, CYP710 clan, CYP711 clan, CYP727 clan, and the CYP746 clan, while the multi-member family clans are the CYP71 clan, CYP72 clan, CYP85 clan, and the CYP86 clan. Notably, the CYP746 clan is exclusive to green algae and mosses, and the CYP727 clan is unique to monocotyledonous plants, with both clans having no known function in dicots [46]. In this study, 233 KcCYPs were identified in *K. coccinea*. Phylogenetic analysis revealed that these KcCYPs belong to 38 families within eight CYP clans, predominantly distributed across the multi-member family clusters of the CYP71 clan, CYP72 clan, CYP85 clan, and the CYP86 clan. The CYP71 clan is particularly significant, accounting for over half of all CYPs in higher plants [46]. Most KcCYPs identified in this study belong to the CYP71 clan, followed by the CYP72 clan, while in *A. thaliana*, the highest gene distribution—apart from the CYP71 clan—is in the CYP86 clan. The distribution of CYP genes in *K. coccinea* is less concentrated in the other five single-member family clans and is absent in the CYP710 clan (Figure 6). Meanwhile, single-member family clans typically encode enzymes with basic functions, whereas multi-member family clans are mainly involved in the secondary metabolism of plants [47].

Cytochrome P450 enzymes (CYPs) are integral to the biosynthesis of diverse natural products, including lignans, through post-structural modifications. Key to this process are the enzymes C3H (CYP98A) and C4H (CYP73A) in the phenylpropanoid pathway, which are crucial for synthesizing coniferyl alcohol, a lignan precursor. C4H, the first identified functional monooxygenase in the CYP family, catalyzes the conversion of cinnamic acid into p-coumaric acid—a reaction initiated by phenylalanine ammonia-lyase (PAL)—which is further processed by C3H and COMT into caffeic acid and ferulic acid, respectively [48,49]. This pathway exemplifies the complex enzymatic processes in lignan biosynthesis. Recent studies have expanded our understanding of CYPs in plant secondary metabolism. Mao et al. [50] demonstrated the integration of two CYPs in tanshinone biosynthesis, highlighting their potential in bioengineering. Awasthi et al. [51] revealed the differential regulation of CYP monooxygenases in flavonoid metabolism under stress in *Coleus forskohlii*, emphasizing CYP’s adaptability in plant defense. The role of CYPs in synthesizing podophyllotoxin, a precursor to anti-tumor drugs like etoposide, has been well-established, with Marques et al. [16] and Lau et al. [26] identifying key enzymes in this pathway. These findings collectively underscore CYPs’ critical role in lignan biosynthesis and their significance in plant metabolism and pharmaceutical development.

The dibenzocyclooctadiene lignans, unique to Schisandraceae plants, were first identified in *Schisandra chinensis* seed oil [52]. Their biosynthesis starts with isoeugenol, which is metabolized to verrucosin and dihydroguaiaretic acid by DIR and PLR enzymes, respectively, before further conversion by OMT, CYP, and UGT enzymes [29]. However, the complete pathway remains to be fully elucidated. In *K. coccinea*, a non-model plant with no complete genome database, the genetic background and biosynthetic pathway of dibenzocyclooctadiene lignans are unclear. Our study focused on the roots, which have a high lignan content [30,53], and identified 62 genes with high or specific expression in roots as candidate CYPs for lignan synthesis. Phylogenetic analysis with reported CYPs involved in lignan synthesis revealed that six homologous genes of CYP719As and one homologous gene of CYP81Qs were highly expressed in roots, suggesting their role in forming methylenedioxy bridges in lignans like gomisin A, schisandrin B, and schisandrin C. Recent studies, such as those by Chen et al. [54] and Qiang et al. [55], have furthered our understanding of dibenzocyclooctadiene lignan biosynthesis in *S. chinensis*, identifying candidate genes and characterizing key enzymes. This ongoing research continues to unravel the complex biosynthetic mechanisms of these pharmacologically significant compounds in Schisandraceae plants, particularly in *K. coccinea*.

The biosynthesis of sesamolin and sesamin, another set of lignans, also involves CYP450 enzymes. CYP81Q1/2 catalyzes the formation of two methylenedioxy bridges in pinoresinol, leading to sesamin, which is further converted to sesamolin and sesaminol by CYP92B14 [27,28]. These enzymes, related to lignan synthesis, predominantly belong to the CYP71 clan. Interestingly, CYP719As and CYP92s are absent in *A. thaliana*, with CYP719As found only in Ranunculales and Aristolochiales and CYP92s in various other plant species [46]. CYP719A1 was the first CYP identified for its role in forming methylenedioxy bridges in alkaloid biosynthesis [56]. However, its homologous enzyme, CYP719A23/24, has been shown to catalyze similar reactions in podophyllotoxin synthesis, despite having only 24% sequence identity with CYP81Q1, which performs a similar function in sesamin synthesis.

*K. coccinea*, belonging to the Schisandraceae family and native to Chinese provinces including Jiangxi, Hunan, Sichuan, and Yunnan, has garnered significant attention in traditional medicine for its diverse pharmacological properties. This plant is particularly noted for its rich content of lignans, with over 121 types identified, predominantly comprising dibenzocyclooctadiene lignans, along with arylnaphthalene and dibenzylbutane lignans [13,14]. The pharmacological efficacy of *K. coccinea*, encompassing anti-inflammatory, liver-protective, anti-HIV, anti-tumor, and anti-aging properties, has been extensively documented in recent studies. Jia et al. [1] highlighted its liver-protective effects, Feng et al. [5] emphasized its anti-inflammatory properties, and Tasneem et al. [7] focused on its anti-tumor potential. Additionally, studies by Liang et al. [8] and Li et al. [9] confirmed its efficacy against HIV and its antioxidant properties, respectively, and Su et al. [10] explored its anti-coagulation effects. Yang et al. [11] contributed significantly by systematically summarizing the phytochemical and pharmacological research on this plant, highlighting its structurally diverse and biologically significant compounds. Recent advancements in understanding lignan biosynthesis have opened new avenues for metabolic engineering to enhance lignan production, as discussed by Satake et al. [22]. The role of microRNAs in regulating lignan biosynthesis, explored by Ražná et al. [23], suggests a complex regulatory network at the molecular level. Behr et al. [19] provided insight into the molecular regulation of monolignol-derived products in hemp, indicating the involvement of dirigent and dirigent-like proteins. Dar and Arumugam [20] reviewed the biosynthesis of sesame lignans, highlighting their health benefits and the involved metabolic pathways. Kim et al. [21] demonstrated the potential of metabolic engineering in *Forsythia* cell cultures for lignan production. Additionally, Kulik et al. [12] investigated the impact of plant lignans on fungal growth and toxin biosynthesis, revealing their broader biological significance. These studies collectively enhance our understanding of lignan biosynthesis and its potential applications in pharmacology and biotechnology.

## 5. Conclusions

In this study, we conducted high-quality transcriptome sequencing and assembly of *K. coccinea* root, stem, and leaf samples, and successfully annotated 158,385 transcripts and 68,978 unigenes using advanced bioinformatics tools. Through comprehensive identification and phylogenetic analysis, we identified 233 CYPs, spanning 38 families across eight clans. Notably, the roots of *K. coccinea*, known for their high lignan concentration, showed specific expression patterns of these genes, suggesting their involvement in lignan synthesis. We identified 22 homologous single genes of these CYPs using sequence alignment analysis, with 6 homologous genes of *CYP719As* and 1 homologous gene of *CYP81Qs* highly expressed in roots, indicating their potential role in forming methylenedioxy bridges in lignans. These results not only advance the understanding of the genetic and biochemical basis of *K. coccinea*, but also contribute significantly to the fields of plant pharmacology and biotechnology. The insights gained from this research pave the way for future exploration into the pharmacological applications of this medicinally valuable plant.

## Data Availability

The datasets presented in this study can be found in online repositories. The names of the repository/repositories and accession number(s) can be found below: https://www.ncbi.nlm.nih.gov/, accessed on 26 January 2024, PRJNA1069246.

## Supplementary Materials

The following supporting information can be downloaded at: https://www.mdpi.com/article/10.3390/genes15030270/s1, Figure S1: Heatmap of Relative Expression of KcCYPs in Different Tissues. The heatmap visualizes the relative expression levels of all KcCYP genes in the roots, stems, and leaves of *Kadsura coccinea*. Blue indicates lower relative expression, while red signifies higher expression levels; Table S1: Specific Primer Sequences of Eight Genes for qRT-PCR; Table S2: Comparative Annotation Statistics of Unigenes Across Multiple Databases.
